# The emergence and implications of SARS-CoV-2 omicron subvariant BA.2.86 on global health

**DOI:** 10.1097/JS9.0000000000001070

**Published:** 2024-01-11

**Authors:** Prakasini Satapathy, Pawan Kumar, Jeetendra K. Gupta, Ali A. Rabaan, Nawal A. Al Kaabi, Dibyalochan Mohanty, Pathakala Naveen, Mahalaqua Nazli Khatib, Shilpa Gaidhane, Quazi Syed Zahiruddin, Ahmad Neyazi

**Affiliations:** a Center for Global Health Research, Saveetha Medical College and Hospital, Saveetha Institute of Medical and Technical Sciences, Saveetha University, Chennai, Tamil Nadu; b School of Pharmacy, Graphic Era Hill University; c School of Applied and Life Sciences, Uttaranchal University, Dehradun, Uttarakhand; d Global Center for Evidence Synthesis, Chandigarh; e EvidenceSynthesis Lab, Kolkata, West Bengal; f Institute of Pharmaceutical Research, GLA University, Mathura, Uttar Pradesh; g Centre for Nano Medicine, School of Pharmacy; h Department of Pharmacology, Anurag University, Hyderabad; i South Asia Infant Feeding Research Network (SAIFRN); j Division of Evidence Synthesis, Global Consortium of Public Health and Research; k One Health Centre (COHERD), Jawaharlal Nehru Medical College, Datta Meghe Institute of Higher Education, Wardha, Maharashtra, India; l Molecular Diagnostic Laboratory, Johns Hopkins Aramco Healthcare, Dhahran; m College of Medicine, Alfaisal University, Riyadh; n College of Medicine and Health Science, Khalifa University; o Sheikh Khalifa Medical City, Abu Dhabi Health Services Company (SEHA), Abu Dhabi, United Arab Emirates; p Department of Public Health and Nutrition, The University of Haripur, Haripur, Pakistan; q Afghanistan Center for Epidemiological Studies; r Academic Affairs, Herat Regional Hospital, Herat, Afghanistan

**Keywords:** BA.2.86, community transmission, COVID-19, epidemiological spread, immune response, omicron, Pirola, SARS-CoV-2, spike protein, vaccine evasion

## Abstract

The SARS-CoV-2 subvariant BA.2.86 ‘Pirola’, first identified in Denmark in August 2023, has manifested with a significantly mutated spike protein profile, suggesting a heightened ability to evade vaccine-induced and infection-induced antibodies. This article outlines the epidemiological spread, immune response implications, and global responses to BA.2.86. Preliminary observations indicate community transmissions of the subvariant, even among those previously infected or vaccinated. Notably, the BA.2.86 infection has shown a potential to amplify antibody responses. The variant’s emergence has evoked memories of the Omicron variant’s rise in late 2021, though global immunity levels might modulate the impact of BA.2.86 impact differently. Continuous genomic surveillance, coupled with integrated diagnostic and epidemiological strategies, proves crucial in early detection and management. The emergence of BA.2.86 reaffirms the unpredictable nature of the COVID-19 pandemic, emphasizing the need for ongoing research, adaptability, and global collaboration.

## Introduction

The COVID-19 pandemic is caused by a virus known as severe acute respiratory syndrome coronavirus 2 (SARS-CoV-2). Since the pandemic began in 2019, the virus has undergone numerous changes, resulting in the emergence of new variants and their subvariants. Each variant carries unique genetic signatures, which can potentially affect its transmissibility, virulence, and potential to circumvent the body’s immune system^1–3^.

The new Omicron subvariant BA.2.86 ‘Pirola’, emerging in Denmark with a heavily mutated spike protein, has swiftly drawn the global scientific community’s focus, particularly in terms of its potential resistance to the vaccines and treatments currently in use, and the broader implications for global public health tactics^4^. This article aims to offer an overview of BA.2.86, examining its characteristics, epidemiological spread, and implications for future pandemic management.

## Origin and characteristics

**Figure 1:**
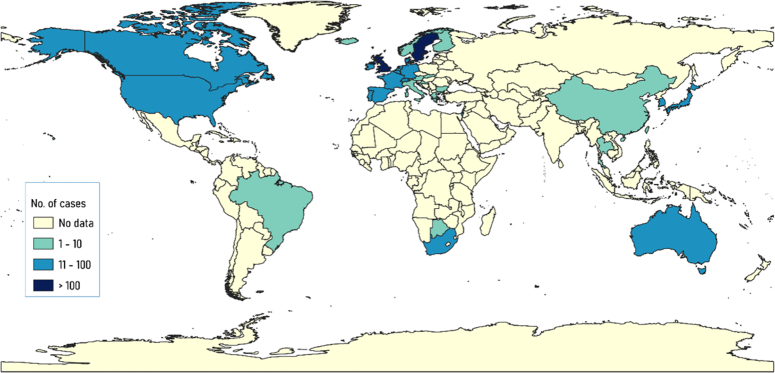
Illustrates the global distribution of SARS-CoV-2 Omicron subvariant BA.2.86 as of 26 October 2023, reported by GISAID (Global Initiative on Sharing All Influenza Data). The figure was generated using QGIS (Quantum Geographic Information System).

First reported in Denmark in August, the BA.2.86 subvariant rapidly expanded to eight countries, with a total of 24 cases identified by the end of the month^5^(Fig. 1). The BA.2.86 subvariant, characterized by a mutated spike protein with 29 substitutions, 4 deletions, and 1 insertion compared to its ancestor, BA.2, indicates a potential increased ability to evade antibodies induced by vaccines and prior infection. Out of the initial cases, none have been severely ill, though the data remains in its early stages^6,7^.

The emergence of BA.2.86 has also drawn comparisons to the appearance of the Omicron variant in late 2021. However, given the broader global immunity resulting from previous infection waves and vaccine rollouts, the impact of BA.2.86 may differ from that of Omicron^8^.

## Evolution of SARS-CoV-2 Omicron

**Figure 2:**
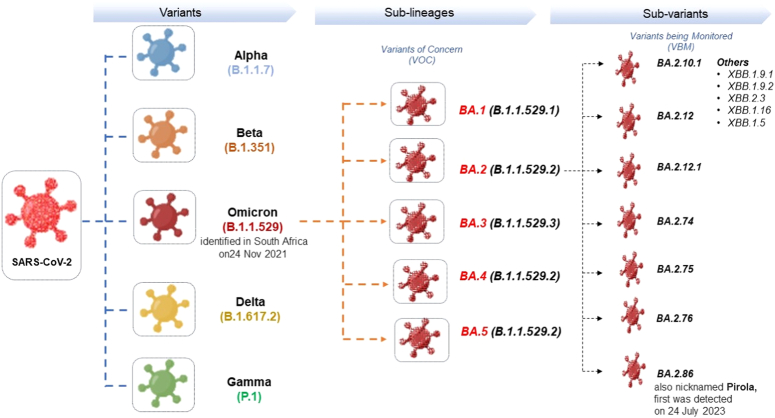
Evolutionary mapping of SARS-CoV-2 variants: focusing on the emergence of Omicron subvariant BA.2.86 ‘Pirola’.

Figure 2 depicts the evolution of the SARS-CoV-2 virus, responsible for the COVID-19 pandemic, which has undergone significant genetic diversification since its emergence^9^. Among its many mutations, a noteworthy development is the evolution of the Omicron variant. The new subvariant, colloquially named ‘Pirola’, represents a continued lineage of the Omicron variant, which is characterized by a series of mutations that distinguish it from its predecessors. While the original Omicron variant was identified in South Africa on 24 November 2021, the Pirola subvariant was first detected several months later, on 24 July 2023. Its emergence underlines the virus’s capacity for mutation and the importance of vigilant genomic surveillance to monitor the evolution of the virus and potentially adjust public health responses accordingly (Fig. 2).

## Transmissibility, infectivity, and immune evasion

Due to its potential impact on transmissibility, infectivity, and immune response evasion, BA.2.86 is causing significant concern. ‘Transmissibility’ refers to how easily the variant spreads from person to person, with higher transmissibility potentially leading to rapid outbreaks and complicating containment efforts. ‘Infectivity’ relates to how effectively the virus can enter and multiply within human cells, which can influence the severity of the disease it causes. The most alarming aspect is ‘immune evasion’, which is the variant’s ability to overcome the defense mechanisms built by previous infections or vaccinations^10^. Variants with improved immune evasion can cause breakthrough infections even in populations that were previously considered protected. The BA.2.86 subvariant, characterized by its heavily mutated spike protein, could significantly influence three critical areas: transmissibility, infectivity, and immune evasion. The spike protein, which is directly linked to infectivity, is essential for the virus’s entry into human cells. Moreover, mutations in this protein may affect not only the virus’s ability to spread but also its capacity to avoid being neutralized by antibodies^11^. Therefore, thoroughly understanding these factors is crucial in evaluating the risks posed by any new variant and in devising effective public health strategies.

## The potential impact on society and economy

The socio-economic impact of BA.2.86 can be substantial. Due to its faster spread compared to previous variants, it could escalate transmission rates, consequently overburdening healthcare systems and depleting resources. This could lead to more hospitalizations, affecting the workforce due to sickness and quarantine rules. Moreover, the emergence of new variants can negatively influence various sectors, leading to economic disruptions. Understanding the genetic makeup and consequences of these subvariants is vital for devising effective public health measures and mitigating their socio-economic impacts^12^.

## Global response strategies

The rapid identification and tracking of new SARS-CoV-2 variants, like BA.2.86, can play a key role in future pandemic management. Collective immunity from vaccinations or natural infections, especially against the Omicron variants, is crucial in limiting the spread of BA.2.86 globally. Ongoing, detailed surveillance of BA.2.86’s evolution is critical to anticipate and respond to potentially more infectious or harmful sublineages^13^.

Denmark has exemplified effective pandemic response by integrating diagnostic and epidemiological strategies, significantly aiding in the early detection of BA.2.86. Wastewater surveillance has also been essential in gauging the variant’s prevalence^4^. The continuous evolution of the Omicron variant has led to a revaluation of treatment strategies to enhance immune protection. Recent studies underscore the role of hybrid immunity in effectively combating both the original SARS-CoV-2 strain and other variants of concern (VOCs). Adapting to the virus’s evolution necessitates a comprehensive approach that integrates vaccine development, antiviral treatments, and ongoing research.

The emergence of BA.2.86 underscores the continuous evolution of the virus, influenced by global immunity levels, vaccination campaigns, and treatment strategies. It is crucial to understand the variant’s potential effects on disease severity, vaccine efficacy, and transmission dynamics to ensure an effective pandemic response^14^.

## Novel treatment strategies

The global health community is emphasizing the importance of continuous genomic surveillance to track the evolution and spread of this subvariant. This surveillance is critical in understanding the variant’s characteristics and its response to existing treatments and vaccines^15^. There is a focus on adapting current vaccine strategies. Given the potential for BA.2.86 to evade immunity from previous infections or vaccinations, researchers are exploring the modification of existing vaccines to enhance their efficacy against this and other emerging variants^16^. In addition to vaccines, the treatment regime for BA.2.86 includes the use of antiviral drugs^17^. Agents like molnupiravir and the combination of nirmatrelvir and ritonavir, which have shown effectiveness against Omicron and its subvariants, are being considered for treatment^18,19^. Ongoing research is essential to fully understand the efficacy of these drugs against BA.2.86 and to develop new therapeutic options. Moreover, the concept of hybrid immunity is gaining attention. This involves combining the immunity acquired from previous infections with the immunity provided by vaccination. Studies suggest that hybrid immunity might offer more robust protection against various variants, including BA.2.86^20,21^.

## Conclusion

The detection of BA.2.86 highlights the ever-evolving challenge posed by SARS-CoV-2. While the long-term implications of this subvariant remain to be fully understood, its emergence underscores the importance of continuous genomic surveillance, global collaboration, and preparedness. The COVID-19 pandemic’s trajectory remains unpredictable, emphasizing the need for adaptability in response strategies and ongoing research efforts.

## Ethical approval

No ethical approval is required.

## Sources of funding

No funding was received.

## Author contribution

All authors have equally contributed.

## Conflicts of interest disclosure

There are no conflicts of interest.

## Guarantor

Ahmad Neyazi.

## Data availability statement

We have not collected any primary data for this research. The authors confirm that the data supporting the findings of this study are available within the article and/or its supplementary materials.
